# Computational approaches for circRNAs prediction and *in silico* characterization

**DOI:** 10.1093/bib/bbad154

**Published:** 2023-05-03

**Authors:** Camilo Rebolledo, Juan Pablo Silva, Nicolás Saavedra, Vinicius Maracaja-Coutinho

**Affiliations:** Center of Molecular Biology & Pharmacogenetics, Department of Basic Sciences, Scientific and Technological Resources, Universidad de La Frontera, Temuco, Chile; Advanced Center for Chronic Diseases - ACCDiS, Facultad de Ciencias Químicas y Farmacéuticas, Universidad de Chile, Santiago, Chile; Centro de Modelamiento Molecular, Biofísica y Bioinformática - CM2B2, Facultad de Ciencias Químicas y Farmacéuticas, Universidad de Chile, Santiago, Chile; Centro de Modelamiento Molecular, Biofísica y Bioinformática - CM2B2, Facultad de Ciencias Químicas y Farmacéuticas, Universidad de Chile, Santiago, Chile; ANID Anillo ACT210004 SYSTEMIX, Rancagua, Chile; Center of Molecular Biology & Pharmacogenetics, Department of Basic Sciences, Scientific and Technological Resources, Universidad de La Frontera, Temuco, Chile; Advanced Center for Chronic Diseases - ACCDiS, Facultad de Ciencias Químicas y Farmacéuticas, Universidad de Chile, Santiago, Chile; Centro de Modelamiento Molecular, Biofísica y Bioinformática - CM2B2, Facultad de Ciencias Químicas y Farmacéuticas, Universidad de Chile, Santiago, Chile; ANID Anillo ACT210004 SYSTEMIX, Rancagua, Chile; Anillo Inflammation in HIV/AIDS - InflammAIDS, Santiago, Chile

**Keywords:** circRNA, circRNA regulation, circRNA-miRNA prediction, bioinformatics

## Abstract

Circular RNAs (circRNAs) are single-stranded and covalently closed non-coding RNA molecules originated from RNA splicing. Their functions include regulatory potential over other RNA species, such as microRNAs, messenger RNAs and RNA binding proteins. For circRNA identification, several algorithms are available and can be classified in two major types: pseudo-reference-based and split-alignment-based approaches. In general, the data generated from circRNA transcriptome initiatives is deposited on public specific databases, which provide a large amount of information on different species and functional annotations. In this review, we describe the main computational resources for the identification and characterization of circRNAs, covering the algorithms and predictive tools to evaluate its potential role in a particular transcriptomics project, including the public repositories containing relevant data and information for circRNAs, recapitulating their characteristics, reliability and amount of data reported.

## INTRODUCTION

The past decade has seen growing interest in the role of circular RNAs (circRNAs) in various diseases [1–5], highlighting their regulatory potential compared with other RNA species [6–8]. circRNAs are single-stranded non-coding RNA molecules that are covalently connected by their 3′ and 5′ ends, generating circRNA molecules rather than the linear structure found in typical RNAs. They can be composed of single or multiple exons, derived from intronic sequences and generated from both long non-coding RNAs (lncRNAs) or messenger RNAs (mRNAs) [1]. These molecules are resistant to exonucleases, leading to a long lifespan of more than 48 hours, which is almost twice the lifespan of mRNAs [9, 10]. Studies have shown that circRNAs are also important endogenous RNA competitors, acting as microRNA (miRNA) sponges, regulating gene expression [11].

circRNAs are present in different subcellular locations [12, 13] and their expression levels change in several tissues during development and cell differentiation [14, 15]. Although circRNAs are poorly conserved across species, apparently arising through convergent evolution, they seem to be more species-specific compared with other RNAs [14]. Literature suggests that transposable elements inserted during evolution have stabilized them and enabled their production [16].

Research has also pointed circRNAs as promising biomarkers for several diseases, including cardiovascular infirmities and cancers. For example, *circANRIL* has been linked to atherosclerosis [17], and *circMICRA* was described as a predictive molecule potentially associated to left ventricular dysfunction [18]. In cancer, *circSMARCA5* has been correlated with tumor differentiation and cancer diameter [19], whereas *circCDYL* has been shown to act as a predictive and prognostic marker of breast cancer [20].

There are a multitude of algorithms for identifying back splicing junctions (BSJ) and circRNA formation. These algorithms are implemented in computational tools with high capacity for predicting these molecules, usually by extracting information from RNA sequencing (RNA-seq) data [21]. Predicted circRNAs are often deposited in databases and repositories that annotate the identified molecules based on various features, such as their genomic coordinates, strand of origin, mature nucleotide sequence, among other characteristics [22]; such as their interactions with other molecules like small RNAs and RNA-binding proteins (RBP). Normally, these circRNA-small RNA or circRNA-RBP relationships are also obtained through computational approaches, which are useful to obtain novel layers of information in their *in silico* characterization and, consequently, obtain further insights related to their functional roles in the cells [23, 24].

In this review, we will first provide an overview of what circRNAs are and how they are originated at the cellular level. This information is crucial for the development of predictive tools for its identification by exploring RNA-seq data or even using the transcript nucleotide sequences features. We will explore the different tools available for circRNA prediction, discussing the positive and negative aspects of them. We will then review the approaches to identify splicing variants and to perform a quantification, normalization and differential expression analysis of these RNAs, as well as the various algorithms for predicting their interactions with other molecules and to obtain insights of their functional roles in the cells. Finally, we provide a brief overview of the databases and repositories containing relevant data and information for circRNAs.

### The biogenesis of circRNAs and key features to be considered in its computational prediction

**Figure 1:**
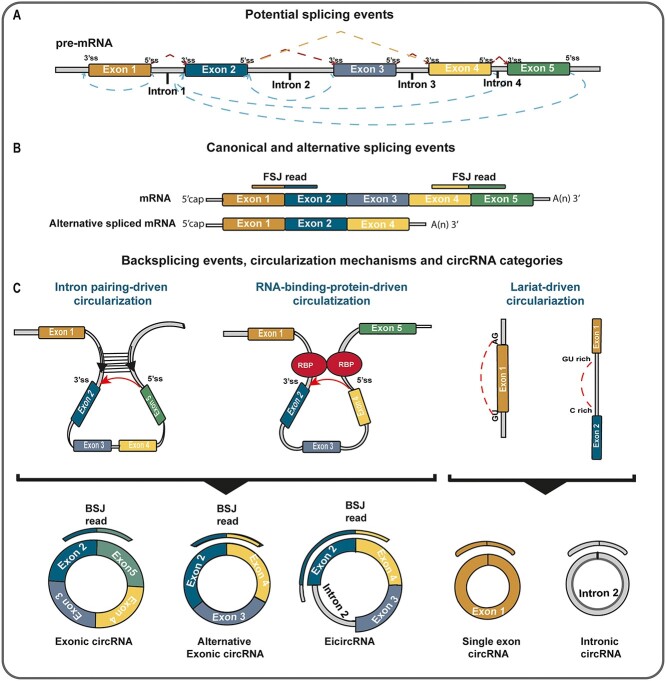
General back-splicing mechanism of circRNAs. (**A**) Transcription mediated by RNA polymerase II gives rise to a pre-mRNA that is subjected to canonical or alternative splicing, becoming mature mRNA accessible to protein translation and detectable as Forward splice junction reads (FSJ reads). (**B**) Intron-free mature mRNA have special well-defined characteristics, including defined 5′ - and 3′ sites, capping and poly(A) tail. (**C**) In circRNAs, alternative splicing is replaced by back-splicing reaction generating a diversity of circRNAs through different mechanisms of circularization such as: Intron pairing-driven circularization facilitated by proximity of donor (5′) and acceptor (3′) sites by inverse complementary sequences near to BSJ splice site, RNA-binding-protein-driven circularization, mediated by proteins that forms dimers allowing to circularize RNA sequence, and lariat-driven circularization facilitated by specific motifs (GU-AG; GC rich zone and others) on flanking sequences. In this manner, different factors bring about a diversity of circRNAs.

The formation of mature mRNAs or lncRNAs is preceded by splicing and rearrangement processes which take place on primary RNAs (Figure 1A). Two different forms of mature RNAs can arise depending mainly on splicing: linear RNAs or circRNAs. The linear mature RNA is generated by the canonical splicing event, in which introns are removed from pre-mRNA and exons are joined together to form a mature mRNA. The spliceosome, a complex of proteins and RNA molecules, recognizes the intron-exon boundaries and removes the introns through a series of enzymatic reactions. Canonical splicing results in a single linear mRNA molecule from each pre-mRNA transcript. Alternative splicing events can also happen in this process, by which different combinations of exons can be included in the mature mRNA, resulting in multiple mature mRNA transcripts from a single pre-mRNA transcript. Alternative splicing is regulated by a combination of *cis*-acting elements in the pre-mRNA and *trans*-acting splicing factors (Figure 1B).

The circRNAs are formed when the cell spliceosome machinery joins the 3′ and 5′ ends of the canonical spliced RNA through a back splicing reaction, which results in the circularization of the RNA molecule (Figure 1C). Aufiero et al. (2019) [25] wrote an extensive review describing the mechanisms of circularization, pointing out three main processes: intron pairing-driven circularization, RNA-binding-protein-driven circularization and lariat-driven circularization. (i) The intron pairing-driven circularization is based on the complementarity of *cis* elements present in flanking introns, which normally are short non-coding regulatory sequences or short interspersed nuclear elements, like Alu sequences and GU-rich sequences, possessing sequence complementarities that guides the covalently bonded structure conformation that facilitates the circularization and production of the mature circRNAs. (ii) RNA-binding protein-driven circularization depends on *trans* elements guided by RBPs that interact with *cis* elements to conform the circularization. These proteins recognize and bind to specific motifs in the intronic flanking regions. In this way, these proteins can dimerize and achieve structures that allow the back-splicing conformation and, consequently, the RNA circularization. Liu et al. (2019) [26] used a neural network model to classify *cis* elements in flanking regions of back-splicing sites of 21,472 human circRNAs, identifying RNA binding and protein binding sites as a main factor for circRNA biogenesis. Huang et al. (2020) [27] summarized the functions of RNA-binding proteins, such as ADAR, QKI and FUS, and nuclear factors like NF90/NF110 as facilitators of this back-splicing process. (iii) In lariat-driven circularization, the intronic or exonic sequences containing a looped structure called ‘lariats’ are removed from the primary RNA during linear splicing, and the lasso-like structure is confirmed by sequence complementarity, giving rise to a circRNA that can be of exonic or intronic origin.

Distinct categories of circRNAs are recognized based on the origin of the genomic region that conforms the final circRNA sequence. Normally, intron pairing-driven and RNA-binding-protein-driven circularizations can generate three main categories, which can include the combination of multiple exons or intronic regions in the final circRNAs (Figure 1C). (i) The exonic circular RNAs (EcircRNAs) includes all exons part of the circularization event; (ii) the alternative EcircRNAs, which includes the exons processed after an alternative splicing event; and (iii) the exonic–intronic circular RNAs (EIcircRNAs), in which an intronic region is retained in the final circRNA together with the exons part of its sequence. Lariat-driven circularization normally generates single EcircRNAs or intronic circular RNAs (IcircRNAs), which involves the base-pairing of splicing sites motif sequences of exonic and intronic sequences, respectively.

These characteristics are important features to be considered in the prediction of circRNAs, and different approaches are being developed based on evaluation of the mapping structure of RNA sequencing reads against a reference genome or even through machine learning approaches considering characteristics present in the sequence of a protein-coding or non-coding transcript.

### Algorithms and computational tools for circRNA prediction

RNA-seq and bioinformatic analysis provide a comprehensive understanding of the eukaryotic transcriptome and a deep exploration of the various types of RNA molecules available in the cell. For circRNA analysis, RNA-seq of total RNA with ribosomal RNA depletion is widely used, often followed by an RNase R step - which efficiently removes linear RNAs - to increase the necessary depth of sequencing. Despite the effectiveness of this approach, results can vary depending on circRNA expression levels, sample quality and sequencing quality [21, 22].

**Figure 2:**
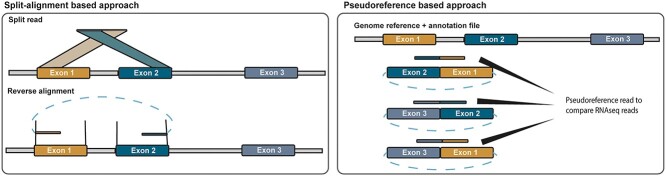
General strategies for circRNA detection. In the split-alignment-based approach, circRNA sequencing reads are split and then reverse aligned to reference genome. In the pseudo reference-based approach, pseudo-sequences are constructed from annotated exons and sequence reads are aligned against the pseudo-sequences.

The software normally developed to explore RNA-seq data can be classified into two main categories, associated with the mapping of sequencing reads against a reference genome and/or gene annotation file: split-alignment-based (segmented-based) and pseudo-reference-based (candidate-based) [28]. Segmented-based tools identify BSJ by aligning reads to the reference genome and are considered *de novo* tools, whereas pseudo-reference tools rely on gene annotation files to identify junction reads (Figure 2). To give more details on how these algorithms are implemented in predictive tools, we chose to briefly describe three of them: CIRCexplorer3 [29], CIRIquant [30] and find_circ [31].

CIRCexplorer3 [30] requires trimmed paired-end FASTQ files from RNA-seq samples, a genome reference and annotation files as input. It first uses HISAT2 [32], a versatile aligner, to map reads to the genome reference, with a focus on finding reads spanning splice junctions in coding transcripts. Unmapped reads are then remapped to the same reference genome. The use of STAR [33], a fast and user-friendly aligner, with an annotation of the reference genome allows the identification of candidate circRNA reads. The precise start and end positions (donor and acceptor sites) of each BSJ event are then determined, and the BSJ is remapped to the annotation file. The final output file contains circRNA annotation information such as the circRNA ID, chromosome, start and end coordinates, strand, read number part of the circRNA, flanking introns and other features interesting for downstream analyses.

CIRIquant [30], the latest version of CIRI2, aligns reads using HISAT2 [32] as the first step. It then makes a *de novo* identification of circRNAs from unmapped reads, which are mapped to the reference genome using BWA [34]. Then, the SAM files are scanned twice. During the first scanning, paired chiastic clipping (PCC) signals are used to detect BSJ and candidate circRNAs; which are then filtered using paired-end mapping (PEM) and splicing junction signals (GT-AG). After grouping BSJ reads and register circRNA candidates, the SAM file is scanned again to detect additional circRNA candidates, and an additional filter is applied to eliminate false positives that are incorrectly mapped to homologous genes and repetitive sequences. The final output file also contains circRNA annotation information such as circRNA ID, chromosome, start and end cordinates, strand, read number, flanking introns and others.

Find_circ [31] is a *de novo* detection algorithm for circRNAs prediction. The input files need to be trimmed to the best possible Phred Quality Score. The first step involves alignment to the reference genome using Bowtie2 [35], with SAM format files resulting from the unmapped reads stored. The second step involves the detection of splicing by extracting 20-mers from both ends of the unmapped reads and aligning them to the reference genome to find unique anchor positions. The output file contains all detected splice junctions in BED format, along with additional columns of information and statistics. In 2018, Hansen [36] published a slight improvement to the find_circ pipeline, which increased the default mapping quality threshold, resulting in a decrease in false discovery rate (FDR).

**Figure 3:**
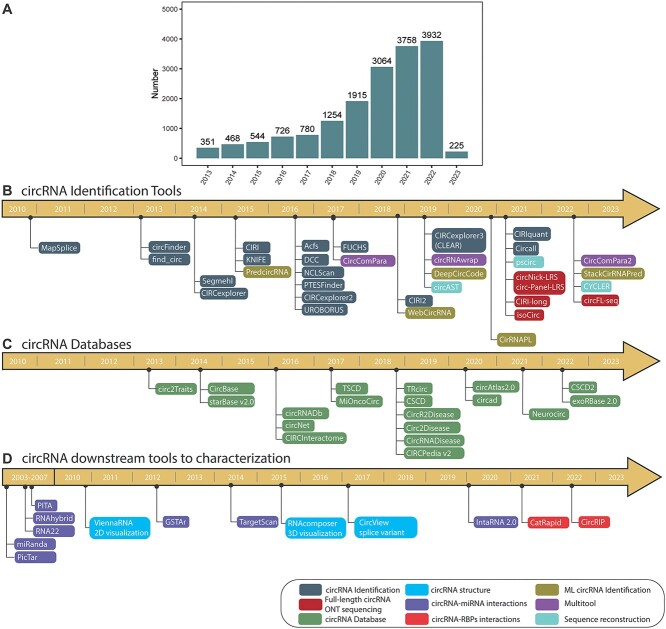
Main circRNA tools in the circRNA research along the last decade. (**A**) Representation of the number of publications on circRNA investigation containing the term ‘circRNA’ in the title or abstract searched in PubMed by year. (**B**) Summary of circRNA identification tools, multi-tools software and sequence reconstruction tools; (**C**) circRNA publicly available databases; and (**D**) other useful tools for characterization, visualization and functional exploration of circRNAs developed in the last decade.

Over the last decade, the interest of the scientific community on circRNAs has grown, and it is reflecting in an increase in more than 90% in the number of publications related to this molecule in literature (Figure 3A). This interest is also reflected in the numerous tools for circRNAs identification, which together with other bioinformatic tools and databases is helping on the further characterization of this new trend in RNA biology research (Figure 3B). Despite the various algorithms and tools developed by bioinformaticians for circRNA analysis, few systematic evaluations of its performance have been carried out [21, 36, 37]. A study by Hansen et al. (2016) [17] showed that five different tools (circRNA_finder [38], MapSplice [39], CIRCexplorer [40], CIRI [41], find_circ [31]) had only a modest overlap of 16.8% in their predictions, highlighting that the circRNA landscape can differ dramatically depending on the algorithm used. It is worth mentioning that 40% of the predicted circRNAs were obtained by only one tool. To evaluate the true positive candidates on this tool-specific group, they treated samples with RNase R and evaluated their performance, concluding that apart from CIRCexplorer [40] and MapSplice [39], more than half of the potential circRNAs were RNAse R sensitive candidates. Thus, revealing to be false positives, suggesting that in most of the cases, the RNAs predicted by several algorithms were likely to be artifacts. To avoid missing potential circRNAs, as well as the identification of false positive artifacts, it is recommended to use more than one algorithm.

Sensitivity and precision are important parameters to evaluate bioinformatic tools performance. In these software, sensitivity refers to the number of reads assigned to each circRNA by the algorithm and is reflected in the total number of circRNAs predicted. On the other hand, precision refers to the accuracy of the algorithm and can be increased by filtering for circRNA candidates by implementing thresholds for read counts. In 2017, Chen and colleagues [37] performed a benchmarking of 11 circRNA detection tools, identifying that NCLScan [42], MapSplice [39], CIRCexplorer [40], DCC [43] and PTESFinder [44] tended to have a low false-positive rates, whereas Segemehl [45], find_circ [31] and UROBORUS [46] yielded the worst performances. In general, NCLScan [42] and CIRCexplorer [40] dominated other tools regarding the precision measuring, whereas KNIFE [47], CIRI [41], Segemehl [45], PTESFinder [44] and CIRCexplorer [40] tended to be more sensitive than other tools. Considering the sensitivity analysis for paired-end data, when both reads span the same BSJ—which can be the case for small circRNAs, different tools undertake distinct results, evidencing an enormous variation of distances between splicing sites engaged in back splicing regions, ranging typically from a few 100 nucleotides to dozens or even hundreds of kilobases.

Later, in 2018, Hansen [36] evaluated the impact of pre-processed reads on algorithm performance, including low-quality read removal and adaptor trimming. In his benchmarking, some algorithms benefited more than others from read trimming, and the sensitivity measured by read count increased modestly, although most algorithms showed unchanged median expression. Most approaches presented a median expression of 14–20 reads, with DCC [43], circRNA_finder [38] and UROBORUS [46] showing the lowest sensitivity with 11, 9 and 5 median reads per circRNA, respectively. It always depends on the initial quality of the RNA sequencing dataset. Recently, Circall [48] has been developed as a tool to specifically identify EcircRNAs from paired-end RNA-Seq data. It boasts improved true positive detection in comparison to CIRI2 [49], CIRCexplorer [40], MapSplice [39] and find_circ [31]. Furthermore, it is faster in analysis, thanks to its implementation of the RapMap aligner [50], which employs a quasi-mapping strategy.

In addition to the two primary methods for identifying circRNAs, Chen et al. (2021) [51] published a review describing machine learning approaches for circRNA prediction. PredcircRNA [52] and WebCircRNA [53] extract features from transcript sequences to predict the formation of circRNAs, whereas DeepCircCode employs a deep learning model to predict back-splicing for the formation of human circRNAs. This tool also incorporates a visualization method to represent circRNA formation features as sequence motifs. CirRNAPL [54] predicts circRNAs based on structural features and composition of sequences, and the recently released StackCirRNAPred [55] classifies circRNAs from other lncRNAs using a stacking strategy. These tools can complement established split-bases and pseudo reference-based identification methods available in the literature. A complete list of circRNA predictive tools was made available in Table 1.

Before start using and testing these tools, it is also important to consider those that are easy and less time-consuming to install and configure. To facilitate the configuration of the proper environment to run a circRNA predictive analysis we recommend to use tools available in Conda environment (https://conda.io/), which is an open-source package management system to facilitate the installation and configuration of software packages written in any language that runs in multi operation systems such as Windows, macOS and Linux.

### Multi-tool approaches for circRNA prediction

Multi-tool approaches for circRNA prediction can address the issue of differences in predicted circRNA numbers and high false positives caused by the difference in algorithm methodologies used by different tools. CircComPara [64] integrates CIRCexplorer [40], CIRI [41] and find_circ [31] to improve the accuracy of circRNA prediction, and its recent version, CircComPara2 [57], integrates CIRI [41], find_circ [31], CIRCexplorer2 [56] (using multiple aligners such as STAR [33], BWA [34], Segemehl [45] or TopHat [65]), further enhancing the identification of true circRNAs. CircRNAwrap [58] is another multi-tool approach that integrates eight circRNA detection tools (KNIFE [47], find_circ [31], CIRCexplorer2 [56], CIRCexplorer [40], MapSplice [39], Acfs [66], circRNA_finder [38] and DCC [43]), three sequence reconstruction approaches (RAISE [67], CIRI-AS [68] and CIRCexplorer2 [56]), and several downstream tools (e.g. Sailfish-cir [69]) for expression abundance estimation and other types of computational characterization.

### Exploring alternative splicing events in the reconstruction of circRNAs

Several computational tools are used to predict circRNAs by looking for BSJ events. However, the internal composition of circRNAs splicing can be diverse and different from the host linear transcripts. The variety of alternative splicing variants requires the comprehensive analysis of circular transcripts. Therefore, to identify circRNA isoforms, the whole coverage of RNA sequencing reads throughout the circular transcripts is necessary. Two major strategies for identifying inner spliced isoforms of circRNAs are CIRCexplorer3 [29] and CIRI-AS [68], part of CIRI package [41, 49].

CIRCexplorer3 [29] uses information on splice-site coverage and depth based on polyA+ and polyA-/RNase R+ reads, whereas CIRI-AS utilizes paired mate reads of back-spliced reads (BSR) to predict forward splice junctions. This last tool categorizes alternative splice sites into: alternative 5′ splice site (A5SS), alternative 3′ splice site (A3SS), exon skipping (ES) or intron retention (IR). However, to run CIRI-AS [68], the paired-end reads must have the same length. It is worth mentioning that CIRI2 [49] can process sequencing reads of different lengths, but CIRI-AS [68] cannot. It is recommended to use raw reads directly or trim all reads to the same length before running BWA [34], CIRI2 [49] and CIRI-AS [68].

Other useful computational methods for the full-length reconstruction and alternative spliced circRNA abundance determination from RNA-seq data include circAST [60], psirc [61] and CYCLeR [62]. circAST [60] is a downstream analysis tool that employs a multiple splice graph method on RNA-seq data and uses upstream circRNA identification tools such as UROBORUS [46], CIRI2 [49] or CIRCexplorer2 [56] to detect back-spliced events. On the other hand, psirc [61] can identify full-length circRNA isoforms and quantify their expression levels from RNA sequencing with results comparable to CIRCexplorer2 [56] and CIRI2 [49]. Recently, the software CYCLER [62] has been developed to identify and reconstruct the predominant isoform of a circRNA with better performance than CIRCexplorer2 [56] and CIRI-full [70].

### Long-read sequencing technologies for the estimation of full-length circRNAs and splicing variants

Ruan et al. (2022) [71] presented a comprehensive overview of different experimental strategies for full-length sequencing splicing variants detection of circRNAs. The authors summarize various library preparation techniques that enrich the sample for circRNAs and pipeline analysis using Oxford Nanopore Technology (ONT) sequencing. These techniques include circNick-LRS [72], circ-Panel-LRS [72], CIRI-long [73], isoCirc [74] and circFL-seq [75]; and their strategies differ in terms of sample preparation and amplification approaches, length of circRNAs obtained and detection of specific features.

The circNick-LRS and circ-Panel-LRS [72] approaches sequence full-length circRNAs through sample enrichment and specific amplification of circRNA transcripts, respectively, followed by ONT sequencing [72]. The circFL-seq [75] and CIRI-long [73] methods use the rolling circle reverse transcription (RCRT) strategy to produce cDNA and detect circRNAs. The circFL-seq assay uses an anchor primer for second-strand synthesis, whereas CIRI-long employs template switching. The isoCirc method [74] utilizes rolling circle amplification (RCA) to enrich BSJ reads, resulting in the detection of circRNAs with sizes ranging from 300–460 nucleotides. circRNA detection using these methods differs in terms of the length of circRNAs obtained. For example, the circNick-LRS [72] approach detects full-length circRNAs with an average size of 795 nt, whereas CIRI-long detects circRNAs less than 500 nt. The circFL-seq approach enables the identification and reconstruction of longer circRNAs up to 2334 nt, and the detection of fusion circRNAs, allowing for isoform-level studies. These approaches also allow for the detection of other specific circRNA sequence features such as microexons inclusion, which are important regulators of the transcriptome [72].

An alternative to explore circRNA splicing variants is to use their identifiers in public databases and look for the mature sequence of circRNA isoforms in repositories like the circAtlas [76], which were reconstructed using the CIRI-full/CIRI-vis pipeline. Unlike linear RNAs, circRNAs have complex and diverse splicing patterns, with many isoforms from a single circRNA. CircView is a useful tool for visualizing circRNA isoforms obtained from various identification tools [77]. High-throughput techniques such as Illumina-based RNA-seq, microarray [40] and NanoString [78] have been used to profile circRNA expression by detecting and counting unique BSJ sequences, but none can detect the full structure of circRNAs >300 nucleotides or determine their exon composition. Alternatively, ONT platform provides comprehensive characterization of alternative splicing events in circRNAs on a genome-wide scale and has the capacity to study sequence modifications in the RNAs, such as the N6-Methyladenosine (m6A), the most abundant internal modification associated with eukaryotic mRNAs [14]. These epitranscriptomic modifications are detected through the direct sequencing of RNA molecules without the necessity of generating a cDNA.

The structural confirmation is also relevant when studying the impact of full-length circRNA isoforms. Secondary structure and thermodynamic parameters can be obtained using the ViennaRNA Package 2.0 [79], considering a particular parameter devoted specifically for circRNAs (‘RNAfold -p -d2 –circ’). Other key tools have been proposed to analyze the superior structures of circRNAs. These include RNAcomposer [80], which can predict the three-dimensional structure of RNA and can be applied to circRNA sequences (http://rnacomposer.cs.put.poznan.pl/), as well as RNAfold from the Vienna package [79] and 3dRNA [81]. These tools can provide new insights into circRNA structures and interactions, offering a deeper understanding of this complex molecule. The use of these tools may reveal new information about circRNA interactions at previously uncharacterized levels.

In summary, transitioning from next-generation sequencing (NGS) to third-generation sequencing (TGS) is a viable and effective option to move forward circRNA characterization, as TGS can sequence full-length reads that provide more information in downstream analyses on the complete circRNA sequence, including the splicing variants, characterization of interaction sites, modifications and superior structures. However, TGS is currently more expensive and has a higher error rate than NGS, but novel improved tools are starting to be released to overcome these limitations.

### Quantification, normalization and differential expression of circRNAs

Once the repertoire of circRNAs available in a transcriptome assay is defined, the next step is to obtain their expression patterns. Quantifying, normalizing and differentiating the expression of circRNAs remains a challenge in transcriptome analysis. CircRNAs, being rare in nature and often displaying low read counts, pose difficulties in obtaining their expression patterns. This is because a small fraction of the total reads from RNA-seq samples support the detection of BSJ, leading to difficulties in differentiating reads from the inner circRNA sequence and the linear form of the transcript. As a result, the number of total sequenced reads can impact the number of identified circRNAs. To mitigate this challenge, researchers have used the previously mentioned RNase R treatment during library preparation, which enriches the sample with circRNAs and focuses the sequencing procedure on circRNA sequences.

Before conducting differential expression analysis, many authors apply a filter to reduce false positives and increase precision of circRNAs identification. For instance, when generating an expression matrix containing each circRNA expression values to be used in a downstream differential expression comparison, studies normally consider only those circRNAs containing read counts greater than 2; or to normalize this expression matrix prior its filtering considering mapped back-splice junction reads per million (RPM) or counts per million (CPM) normalized values greater than 0.1 [49].

Different challenges, such as the number of total read sequences, low read counts for each circRNA, high variability between samples and a lack of coverage modeling, can affect differential expression analysis. Some commonly used software for mRNA differential expression, such as DESeq2 [82] and edgeR [83] can be applied to perform differentially expression analysis for circRNAs. CircTest [43] is another approach that can also be employed. It models the data using the beta binomial distribution and performs an ANOVA to identify circRNAs that differ in their relative expression between groups. Other authors use log2(CPM) representation and perform a Mann–Whitney *U* test to differentiate the expression values of circRNAs within different groups of samples.

### Functional prediction of the biological context of circRNAs

CircRNAs have been described to exert their effects through several mechanisms [84]. One of the main mechanisms is their ability to modulate gene expression by acting as a miRNA and protein regulator [2, 85]. They act as a sponge for miRNAs, ‘capturing’ them through microRNA responsive element (MRE) sites, thereby reducing miRNA availability and influencing mRNA expression levels. Additionally, circRNAs have the potential to modulate alternative splicing or transcription by controlling protein expression and avoiding mRNA transcription. Another mechanism involves circRNAs regulating the parent genes by interacting with RNA pol II, altering its affinity for adjuvants and influencing gene transcription [1]. Finally, some circRNAs have been found to contain open reading frames (ORF) or internal ribosome entry sites (IRES), which have the potential to encode proteins [86, 87]. This is a relatively unexplored area that could have a significant impact at the cellular level.

Computational approaches can be used to predict the interaction between circRNAs and miRNAs or RNA-binding proteins. These predictions can be used to generate interaction networks, by visualizing the results using tools such as Cytoscape [86] or R packages like VizNetwork (https://datastorm-open.github.io/visNetwork/) or networkD3 (http://christophergandrud.github.io/networkD3/), and integrating data from public protein–protein interaction databases such as STRING [88], IntAct [89] or BIND [90]. Enrichment analysis can also be performed through tools such as enrichR [91], PANTHER [92] or DAVID [93], to identify overrepresented biological pathways and processes according to the coding genes present in the generated network.

### CircRNA-miRNA interaction tools

Multiple tools are used to analyze circRNA-miRNA interactions, contributing in the prediction of gene expression patterns on which this interaction could have some kind of effect, favoring its degradation, or changing expression patterns of miRNA and mRNAs through competing endogenous mechanisms. Among them, we can highlight RNAhybrid [94], GSTAr [95], miRTarget [96], TargetScan [97], miRanda [98], PicTar [99], PITA [100], IntaRNA [101], RNA22 [102] and others. These tools have the potential to identify possible MRE sites within circRNAs. In this way, Riffo-Campos et al. 2016 [103] evaluated the possibility of interaction through numerous parameters depending on the tool used. RNAhybrid [94], GSTAr [95], miRTarget [96] perform this analysis by calculating the minimum free energy hybridization between sequences, looking for the most favorable interaction. TargetScan [97] is the tool with better performance, but with a high false negative rate, whereas miRanda [98] has a better sensitivity but with a higher false positive rate. It runs an algorithm based on complementarity, binding energy and taxonomic conservation of the predicted interaction; PicTar aligns seed regions given their high conservation, allowing predicted interactions, and classifies it taxonomically. Finally, PITA [100] calculates the differences between free energies in pairing and unpairing the target binding site to predict the structural accessibility of the seed matching.

### CircRNA-protein interaction tools

Main circRNAs and RBP (RNA-binding protein) interactions are involved in the regulation of gene transcription and translation of peptides. Although RBPs can bind both linear RNAs and circRNAs, evidence suggests a preference for RBP–circRNA binding due to the tertiary structures of circRNAs [104]. RBPs play a role in the modulation and control of circRNA biogenesis [105], whereas, at the same time, circRNAs can serve as ‘sponges’ for RBPs, altering the translation of other proteins [106, 107].

Protein-circRNA interactions are primarily studied through experimental techniques, and specific circRNA tools are limited. Most bioinformatics tools also do not consider the tertiary structure of circRNAs when determining protein-circRNA interactions [27]. A notable online tool is catRAPIDv2.0 (http://service.tartaglialab.com/page/catrapid_omics2_group) [108], which evaluates the propensity for circRNA-RBP interactions by analyzing the circRNA sequence with a fragmentation approach against precompiled RBP libraries from several organisms. Advances in this field mainly focus on either predicting the circRNA-binding sites on the protein, or conversely, the protein-binding sites along the circRNA sequence. In the first case, CircInteractome [109] provides some tools, but it does not consider the full-length sequence of circRNAs. Conversely, there are approaches based on neural networking [110, 111], in which the interaction matrices of known protein-circRNA pairs are used to train a neural network to generate improved prediction models. CRIP [110, 111] is a recent deep learning method that uses known sequences and is based on codon splitting to predict interactions. Another novel method, iCircRBP-DHN [112], uses deep hierarchical networks for circRNA-RBP binding site discrimination. circRIP [113], another recently developed algorithm, is an efficient tool for identifying genome-wide circRNA-RBP interactions from RNA immunoprecipitation sequencing (RIP-Seq) and enhanced cross-linking immunoprecipitation (eCLIP) data, optimizing the quantification process.

### Experimental procedures for validation

The validation of circRNA presence and functionality is crucial in the study of these non-coding RNAs. RT-qPCR is a sensitive method for detecting the expression levels of circRNAs and comparing them to those of linear RNAs. It can be used to quantify the expression levels in different samples, such as normal and diseased tissues, and to monitor changes in response to various stimuli. RT-qPCR of RNase R-treated samples can also be used to validate the detection of circRNAs. The technique requires divergent primers that target the BSJ, a unique sequence within the human genome [114]. RNase R is a 3′-to-5′ exoribonuclease that degrades linear RNAs, but most circRNAs are resistant to degradation due to their closed circular configuration. To confirm the circular configuration, Northern blot analysis, an RNase R-independent technique, is considered a gold standard. Probes are designed to either span the BSJ or hybridize to the total transcript, and by choosing a suitable gel electrophoresis system (agarose and/or polyacrylamide) the two possible configurations (circular or linear) can be clearly distinguished. Northern blotting can also be combined with RNase R or RNase H ribonuclease digestion. The RNase H cleavage assay has emerged as an elegant method for circRNA validation. Antisense oligonucleotides are designed to target a specific circRNA, and RNAse H recognizes DNA–RNA hybrids and cuts the RNA within the duplex. The characteristic shift in mobility during denaturing polyacrylamide gel electrophoresis from the aberrantly slow migration of the circRNA to the expected linear behavior, as well as the cleavage patterns of RNase H digestion, can then be analyzed by Northern blotting [115].

**Table 1:** Different tools for identifying circRNAs and associated methodology.

Identification Tools	Method	Aligner	Aligner strategy	Link	Reference
CIRCexplorer3 (CLEAR)	S.A.B	HISAT2;STAR	Splice-aware	https://github.com/YangLab/CLEAR	[29]
CIRCexplorer2	S.A.B	STAR	Splice-aware	https://github.com/YangLab/CIRCexplorer2	[56]
CIRIquant	S.A.B	HISAT2;BWA	Versatile	https://github.com/bioinfo-biols/CIRIquant	[30]
CIRI2	S.A.B	BWA	Versatile	https://sourceforge.net/projects/ciri/files/CIRI2/ https://ciri-cookbook.readthedocs.io/en/latest/CIRI-full.html#installation	[49]
Find_circ	S.A.B	Bowtie2	Versatile	https://github.com/marvin-jens/find_circ	[31]
KNIFE	P.R.B	Bowtie2	Versatile	https://github.com/lindaszabo/KNIFE	[47]
DCC	S.A.B	STAR	Splice-aware	https://github.com/dieterich-lab/DCC	[43]
UROBOROS	S.A.B	Bowtie	Versatile	https://github.com/WGLab/UROBORUS http://uroborus.openbioinformatics.org	[46]
NCLScan	P.R.B	BWA	Versatile	https://github.com/TreesLab/NCLscan	[42]
MapSplice	S.A.B	Bowtie	Versatile	https://github.com/ahcarpenter/mapsplice http://www.netlab.uky.edu/p/bioinfo/MapSplice2	[39]
PTESFinder	P.R.B	Bowtie	Versatile	https://github.com/osagiei/pfv2	[44]
Segemehl	S.A.B	Internal	Covariance Models	https://www.bioinf.uni-leipzig.de/Software/segemehl/	[45]
CircCall	P.R.B	RapMap	Quasi-mapping	https://github.com/datngu/Circall.	[48]
CircComPara2	S.A.B and R.R.B	Multi-tool	Multi-tool	https://github.com/egaffo/CirComPara2	[57]
circRNAwrap	S.A.B and R.R.B	Multi-tool	Multi-tool	https://github.com/liaoscience/circRNAwrap	[58]
PredcircRNA	M.L	NA	NA	https://github.com/xypan1232/PredcircRNA	[52]
WebCircRNA	ML	NA	NA	https://rth.dk/resources/webcircrna/download	[53]
DeepCirCode	M.L	NA	NA	https://github.com/BioDataLearning/DeepCirCode	[59]
CirRNAPL	M.L	NA	NA	http://server.malab.cn/CirRNAPL/	[54]
StackCirRNAPred	M.L	NA	NA	https://github.com/xwang1427/StackCirRNAPred	[55]
circAST	S.R	-	-	https://github.com/xiaofengsong/CircAST	[60]
psirc	S.R	-	-	https://github.com/Christina-hshi/psirc	[61]
CYCLeR	S.R	-	-	https://github.com/stiv1n/CYCLeR	[62]
CIRI-AS	S.R	-	-	https://sourceforge.net/projects/ciri/files/CIRI-AS/	[63]

Method column abbreviations: S.A.B: split-alignment-based; P.R.B: pseudo reference-based; M.L: machine Learning. S.R.: sequence reconstruction.

### Publicly available repositories for circRNA research

circRNAs are attracting increasing attention from the scientific community and, as a result, a plethora of databases have been established to store extensive information on these RNA molecules (Figure 3C). These repositories provide information such as circRNA sequences, functional predictions, the circRNA–miRNA–mRNA axis and links to diseases [2, 116]. Vromman and colleagues (2020) [18] reviewed these databases and categorized them as curated and non-curated.

In our review, we will address some considerations one should consider when using a repository as a reference. For instance, not all databases use the same genomic coordinates (chr:start-end), which can result from differences in the input file formats used by the databases. BED format-based databases are 0-based, whereas SAM format-based databases are 1-based, thus requiring adjustments when comparing data between databases. Additionally, some repositories may not provide complete information on their data, such as RNA-seq library type (e.g. single-end, paired-end, stranded, RNase R enriched), primary nucleotide sequence of the mature circRNA, strand of origin (e.g. positive, negative), validation methods (e.g. qPCR, long-read sequencing, Northern blot, etc.). This information is crucial because the presence of an exon junction does not necessarily indicate RNA circularization. For example, RNA-seq poly (A) + decreases sensitivity to detect circRNAs as in general these molecules are not polyadenylated, increasing the quantity of linear RNAs. Nevertheless, studies have shown that even RNA-seq polyadenylated libraries can detect quantifiable amounts of exon junctions, suggesting that their presence alone is not enough to ensure circularization [12, 117]. Another example is related to the presence of Alu sequences in the 3′- and 5′- regions of intronic segments [9, 118], or sequence motifs in the flanking regions of the splicing candidates [117]. All of them are also important features to be considered in the determination of the RNA circularization, and the lack of information makes it difficult to validate and curate the candidates available in these repositories. It is worth mentioning that this kind of mechanism remains poorly understood and it is fundamental to have as many details as possible in the metadata indexed in databases.

**Table 2:** Publicly available databases devoted to circRNAs

Database	Type of Daya	Organism	Link	Reference
circAtlas	Tissue-specific circRNAs Evolutionary conservation Regulatory network Full-length sequence of circRNAs	*Gallus gallus Homo sapiens*, *Macaca mulatta*, *Mus musculus*, *Rattus norvegicus*, *Sus scrofa*,	http://159.226.67.237:8080/new/index.php	[76]
circBase	circRNA expression level Sequence	*C. elegans*, *Drosophila melanogaster*, *H. sapiens*, *Latimeria chalumnae, M. musculus*	http://www.circbase.org	[120]
CIRCpedia v2	circRNAs expression in cell types and tissues from different species	*Caenorhabditis elegans, Danio rerio, D. melanogaster, H. sapiens*, *M. musculus*, *R. norvegicus*,	http://yang-laboratory.com/circpedia/	[127]
MiOncoCirc	circRNA associated to cancer	*H. sapiens*	https://mioncocirc.github.io	[121]
CIRCinteractome	Identification of MRE sites Divergent primers and siRNA design	*H. sapiens*	https://circinteractome.nia.nih.gov	[109]
StarBase v2.0/ENCORI	RNA–RNA interactions	23 species	https://starbase.sysu.edu.cn	[128]
CircRic	circRNAs associated to cancer	*H. sapiens*	https://hanlab.uth.edu/cRic/	[122]
Circ2Disease	circRNA associated to diseases	*H. sapiens*	http://bioinformatics.zju.edu.cn/Circ2Disease/index.html	[125]
CircR2Disease	circRNA associated to diseases	*M. musculus, R. norvegicus, H. sapiens*	https://bio.tools/circR2Disease	[129]
circBank	circRNA interactions	*H. sapiens*	http://www.circbank.cn	[130]
Circ2Traits	circRNA-miRNA-disease associations	*H. sapiens*	http://gyanxet-beta.com/circdb/	[119]
CircNet	Tissue-specific circRNA expression patterns circRNA–miRNA–mRNA axis	*H. sapiens*	https://awi.cuhk.edu.cn/~CircNet/php/index.php	[131, 132]
circRNADb	Exonic circRNAs Protein coding-circRNAs annotation	*H. sapiens*	http://reprod.njmu.edu.cn/cgi-bin/circrnadb/circRNADb.php	[133]
CSCD2	circRNAs in cancer miRNA–circRNA interaction RBP-circRNA interaction ORF in circRNAs	*H. sapiens*	http://geneyun.net/CSCD2/	[134]
TSCD	Tissue-specific circRNA expression circRNAs in organogenesis	*M. musculus, H. sapiens*	http://gb.whu.edu.cn/TSCD	[135]
circad	circRNAs associated with diseases	*M. musculus, H. sapiens, R. norvegicus, S. scrofa*	Rophina et al., 2020 https://clingen.igib.res.in/circad/	[123]
TRcirc	Transcriptional regulation of circRNAs	*H. sapiens*	http://www.licpathway.net/TRCirc/view/index	[136]
circRNADisease	circRNA associated to diseases	*H. sapiens*	http://cgga.org.cn:9091/circRNADisease/	[124]
exoRBase 2.0	Extravesicular long RNAs	*H. sapiens*	http://www.exorbase.org	[137]
NeuroCirc	circRNAs in human brain	*H. sapiens*	https://voineagulab.github.io/NeuroCirc/	[138]

Circ2Traits [119] was the first database to provide information on circRNAs, specifically manually curated circRNA-miRNA-disease associations in human datasets. CircBase [120] followed as the second repository published, providing information on expression levels and sequence information of circRNAs from human, fly, mouse, coelacanth and *Caenorhabditis elegans*. It has been widely used as a reference in annotation pipelines. CircAtlas [76], another widely used database, relies on the integration of four tools (CIRI2 [49], CIRCexplorer2 [56], DCC [43] and find_circ [31]) to annotate data from human, macaque, mouse, rat, pig and chicken and provides information on tissue-specificity, evolutionary conservation, regulatory networks, disease associations and full-length circRNA sequences. CircAtlas [76] MiOncoCirc [121] and CircRic [122] are repositories focused on cancer-related circRNAs, analyzing biogenesis, transcriptional landscape, integrative analysis and drug response (exclusive to CircRic). Other disease-related databases contain data on various pathologies, such as cardiovascular infirmities, diabetes, immune disorders, etc. Main resources in this category are those with manual curation, such as circad [123], circRNADisease [124], Circ2Traits [119] and Circ2Disease [125].

Each of the publicly available databases (Table 2) has unique features that support circRNA research [2]. However, one of the key challenges within these resources is the lack of a universal naming convention. For instance, some databases use ‘circ_+ host gene + variant’ or ‘circ_ + ID’ when naming the RNAs, which makes it difficult to compare data between databases for a specific circRNA. To address this issue, Chen et al. (2023) [126] proposed a nomenclature for reporting circRNAs to repositories. This involves naming every circRNA with a ‘circ’ prefix, followed by the parental gene name and the number of the exons, e.g. ‘circGENEY(1,3,5)’ for a host gene of name GENEY in which the exons 1, 3 and 5 conformed the circRNA. If an intronic region is retained, the suggested nomenclature includes an ‘RI’ indicating it, such as ‘ciercGENEY(1,RI,3,5)’. This will help to standardize identification and annotation, as well as enable the sharing and cross-reference of information between databases. It is important for researchers to adopt this nomenclature and publish their data in a format that is consistent with this standard.

In summary, the various circRNA databases have unique characteristics that aid in the study of these RNA molecules. However, there are also challenges that need to be addressed, such as the lack of a universal naming convention, the incompleteness of data in some databases, and differences in genomic coordinates used by different databases. Efforts should be directed toward standardizing these databases to enable more comprehensive and accurate analysis of circRNAs.

## CONCLUDING REMARKS

CircRNAs are a growing class of non-coding RNAs that are increasingly being recognized for their role in cellular processes. Despite the availability of various bioinformatic tools to identify and quantify circRNA expression, the lack of a uniform nomenclature system and the diverse expression patterns across different species, genes, tissues and developmental stages still present challenges in circRNA research. The mechanisms of circRNA action, including sponge effect, interaction and peptide coding, are the focus of ongoing studies. However, the limitations of current bioinformatic approaches hinder a comprehensive analysis of circRNAs, such as the inability to accurately quantify expression levels based on the full circRNA sequence and to consider the mature isoform sequences derived from the same BSJ. Direct RNA sequencing or TGS techniques can address these issues and improve the prediction of miRNA and RBP interactions. Most algorithms for predicting circRNA-protein interactions are based on general properties of non-coding RNAs and overlook the tertiary structures of circRNAs, which can greatly influence these interactions. Although some neural network-based algorithms consider these structures, they require a large amount of training data that is currently limited. To advance circRNA research, the development of specific algorithms and better integration of data is necessary. Additionally, a common nomenclature system is crucial for comparing circRNA expression across different conditions, diseases or tissues in standardized ways within different publicly available databases.

It is worth noting that other publications in the literature have reviewed different aspects of the computational tools for circRNA identification and its in silico functional characterization (see [51, 71, 139]). However, unlike other reviews, our work offers a more accessible description of the most popular tools for circRNA identification, including technical aspects, as well as a compilation of the most popular tools for its in silico characterization in order to obtain functional insights of their biological roles. We aimed to cover various aspects of the entire pipeline for circRNA characterization, starting from the reconstruction of the nucleotide sequence to the prediction of interactions with miRNAs and RBPs, and the obtention of potential ORFs generated by that transcript. Additionally, we covered other aspects related to the study of secondary and tertiary structures associated with the predicted circRNAs, and also emphasized the importance of using new sequencing technologies and the preparation of different types of sequencing libraries to obtain the complete sequence and other characteristics of circRNAs, followed by their validation through molecular biology and biochemical experiments, which allow us to characterize their subcellular location, the integrity of their sequence and their interaction with predicted cellular elements. Our aim is to provide an integrative and multidisciplinary vision to new researchers interested in delving into the biology and bioinformatics of circRNAs.

**Key Points**

- Bioinformatic tools for circRNA prediction and databases should be implemented in an integrative way, to facilitate experimental circRNA-related data interpretation.
- circRNA identification experiments from RNAseq expression data can be evaluated by several detection methods and is case dependent on the implementation of more than one.
- circRNAs have a great potential to regulate other RNA species, and circRNA functions can be explored by interaction prediction pipelines with both miRNAs and RBPs, and should be corroborated by experimental procedures.

## Data Availability

No datasets were generated or analyzed during the elaboration of this work.

## Author Biographies

**Camilo Rebolledo** is a PhD student in Applied Cellular and Molecular Biology at the Universidad de La Frontera, Chile. His research interests are bioinformatics, transcriptomics and molecular biology.

**Juan Pablo Silva** is a PhD student in Biochemistry at the Universidad de Chile, Chile. His main research interests are transcriptomics, circRNAs functional characterization and RNA epitranscriptomics.

**Nicolás Saavedra** is an associate professor and the President of the Scientific Ethics Committee of Universidad de La Frontera, Chile. His research interests are genetics, cardiovascular medicine and microbiome associations with chronic diseases.

**Vinicius Maracaja-Coutinho** is an associate professor at the Universidad de Chile. He leads the Laboratory of Integrative Bioinformatics and is the Director of the Graduate Diploma in Bioinformatics and Computational Biology. His interests are RNA bioinformatics, long non-coding RNAs and RNA epitranscriptomics.
